# Knowledge, Attitudes, and Practices (KAPs) of Farmers on Foot and Mouth Disease in Cattle in Baghlan Province, Afghanistan: A Descriptive Study

**DOI:** 10.3390/ani11082188

**Published:** 2021-07-23

**Authors:** Arash Osmani, Ihab Habib, Ian Duncan Robertson

**Affiliations:** 1 School of Veterinary Medicine, College of Science, Health, Engineering and Education, Murdoch University, Perth 6150, Australia; i.robertson@murdoch.edu.au; 2 Department of Veterinary Medicine, College of Food and Agriculture, United Arab Emirates University (UAEU), Al Ain P.O. Box 15551, Abu Dhabi, United Arab Emirates; 3 Hubei International Scientific and Technological Cooperation Base of Veterinary Epidemiology, Key Laboratory of Preventive Veterinary Medicine, Wuhan 430070, China; 4 College of Veterinary Medicine, Huazhong Agricultural University, Wuhan 430070, China

**Keywords:** KAP, Baghlan, Afghanistan, farmers, animal traders, One Health, FMD

## Abstract

**Simple Summary:**

Foot and mouth disease (FMD) affects the productivity and health of several animals species, including cattle. In Afghanistan, cattle represent a valuable source of food security and play a vital role in the rural economy. Using a questionnaire-based approach, we evaluated the self-reported knowledge, attitudes, and practices of various stakeholders involved in the cattle industry and veterinary management of animal health in a northern province of Afghanistan. The study pointed to several aspects that could be translated into practical management options to add value to FMD management in the cattle industry in Afghanistan.

**Abstract:**

This study was performed to investigate the knowledge, attitudes, and practices (KAPs) of farmers, animal traders, and veterinary professionals on FMD in Baghlan province, Afghanistan. Four structured questionnaires were administered to the respondents. Almost half (48.5%) of the farmers had heard of the occurrence of FMD in their neighbourhood or knew the name of the disease. The majority of farmers could recognise the clinical signs of FMD in their animals (salivation, 85.9%; tongue ulcers, 78.8%; gum lesions, 78.2%; hoof lesions, 76.8%). Most farmers stated that the “introduction of new animals” was the primary cause of FMD appearing on their farms and to control the spread of the disease, over half of the farmers (56%) preferred not to buy cattle from unknown or potentially infected sources. Animal traders’ knowledge was limited to recognising some clinical signs of the disease such as: salivation, and lesions in the mouth and on the feet. No animals were directly imported by the traders from outside Afghanistan. Over half of the local veterinary professionals (65%) kept record books of the animal diseases seen and/or treatment plans undertaken, and 80% of them reported the occurrence of FMD to the provincial, regional, and central veterinary authorities. No regular vaccination programme against FMD was implemented in the province. Poor import controls and quarantine were considered to be the main barriers to the control of FMD in the study area and the surrounding provinces. It can be concluded that, despite relatively good knowledge about FMD in the study area, there are gaps in farmers’ and traders’ knowledge that need to be addressed to overcome the burden of the disease in the province. These should focus on strengthening interprovincial quarantine measures and implementation of regular vaccination campaigns against the circulating FMDV within the area.

## 1. Introduction

FMD is a former List A World Organisation for Animal Health (OIE) transmissible disease that has the potential for rapid international spread, resulting in serious socio-economic consequences and disruption of international trade. Foot and mouth disease is a highly contagious disease with the potential to cause severe economic loss in susceptible cloven-hoofed animals [1,2].

Collecting information relating to animal infectious diseases from farmers is a significant step towards the control and eradication of diseases such as FMD. In low-income countries, such as Afghanistan, livestock, especially dairy cattle, are important and minimising the impact of disease on these animals is critical for maintaining the livelihood of the rural communities. It is important to understand the knowledge, attitudes and practices (KAPs) of farmers when developing and implementing disease control and prevention strategies [3]. Importantly, to ensure that the public, especially the farming community, is aware of the disease, communication between the veterinary authorities and farmers is essential. Since FMD is endemic in Baghlan province, Afghanistan [4], a successful preventive and controlling strategy in this area relies not only on a high adoption level of vaccination, but also on effective responses to outbreaks, restricting and monitoring the movement of susceptible animals within the area [5,6].

FMD has been endemic in Afghanistan for many years. The surveillance system currently adopted in Afghanistan is primarily passive [4,7]. To our knowledge, there have been no studies on the KAP concerning outbreaks of FMD in Baghlan province or other parts of Afghanistan. Therefore, the research reported in this manuscript was developed to assess the extent of the knowledge and understanding of FMD by smallholder dairy farmers and animal traders in Baghlan province and to identify practices at the local farm/household level that potentially result in the disease remaining endemic within the study area. This study was designed to: (1) describe the level of awareness of farmers and traders of FMD; (2) describe the perceptions of the local veterinary professionals (veterinarians, para-veterinarians also known as veterinary assistants and basic veterinary workers) on the disease in the study area; and (3) to provide data that could be used in future disease control programs on FMD in this area.

## 2. Materials and Methods

### 2.1. Study Setting and Questionnaire Interview Procedure

Given the fragility of the security situation of Baghlan province, the three districts of Khinjan, Doshi and Puli Khumri were selected for operational safety and convenience. These districts have sizable livestock populations (especially ruminants), a history of FMD being present based on reports by local farmers and veterinary authorities, and a large number of animal movements entering and exiting. Additionally, there is a major highway (Kabul-North highway or “Ring Road” which is the only trans-Hindukush highway) passing through these districts which connects cities in the central part of Afghanistan, including Kabul, the capital city, to the northern and north-eastern provinces of the country. A total of 53 villages within the three districts were selected based on safety, proximity to the Kabul-North highway with good road access and a large cattle population. Within the selected villages, 198 households/cattle-herds were randomly selected from a list of all herds (*n* = 450) in these villages provided by local veterinary professionals (there are no official records of the exact number of herds present in the surveyed villages, however the local veterinary authorities, based on their experience, estimated there were 450 herds present in these villages). Details of the study setting, field methodology, and epidemiological terminology that describe the animal husbandry and veterinary services provided to the farmers in the study area were previously been explained in Osmani et al. [8]. A questionnaire was administered at the same time as serological and socio-economic studies were performed. All farmers were interviewed at their homes. Prior to administering the questionnaire, the purpose of the study was outlined, a brief explanation of FMD was provided and oral consent to participate in the study was obtained. The questionnaire interview was applied to the cattle farmers by the first author and a team of local veterinary professionals (*n* = 8).

Questionnaires for farmers: A questionnaire, approved by the Murdoch University Human Ethics Committee (2017/004), was administered to 198 livestock owners in a face-to-face mode. Questionnaires were administered in the early morning or late afternoon to each farmer at their home by one veterinarian and one para-veterinarian over 16 days (5 to 20 May 2017). At the start of the interview, the respondents were advised of the survey’s objectives; oral consent to undertake the questionnaire was obtained from the farmers; and a brief explanation about FMD was provided. The questionnaire was designed to assess the farmers’ KAP regarding FMD in their herds, in particular focusing on the disease in cattle. Farmers were shown photographs of FMD lesions during the questionnaire interview.

Questionnaire for animal traders: Twenty-five local animal traders from Baghlan province were included in the survey to determine the practices adopted and their perceptions towards FMD during the routine trade of livestock/cattle.

Questionnaire for veterinary professionals: Twenty local veterinary professionals and 30 senior veterinary professionals from Baghlan province and surrounding provinces were included in this survey to determine the practices adopted and their perceptions towards FMD in the area. Experienced veterinary professionals from surrounding provinces were selected for inclusion in this study with the assistance of staff from the Veterinary General Directorate of the Ministry of Agriculture, Irrigation and Livestock of Afghanistan. Two main areas were covered (perceptions about FMD, and potential reasons for the failure to control FMD) in this questionnaire. To study the experienced veterinary professionals’ perception of infectious diseases, specifically FMD, this cohort was asked to score each question from 1 to 12 based on its importance. The answers were then divided into three categories of: most important with a score of 1 to 4; moderately important with a score of 5 to 8; and least important with a score of 9 to 12. The questionnaire for veterinary professionals in surrounding provinces was administered either through email (*n* = 5) or through social media (Facebook messenger *n* = 25). All the interviews and questionnaires were conducted in the local language (Dar/Farsi or Pashto). The questionnaire is available from the corresponding author upon request.

### 2.2. Data Analyses

Data obtained from the questionnaires were entered into an excel worksheet (Microsoft^®^ Excel for Mac, 2017). Data management and analyses were carried out in MS Excel and Statistical software R [9]. Descriptive statistics were generated for each variable of interest. The percentages and their 95% confidence intervals (95% CI) were calculated to determine the extent of the participants’ knowledge and practices towards FMD in their herds/study area and in surrounding provinces. Questions on knowledge were used to determine the participants’ (farmers and animal traders) general knowledge regarding the disease, its clinical signs, and modes of transmission. Questions on attitudes and practices were used to assess farmers, traders and veterinary professionals’ perceptions on control measures and disease prevention methods. The questionnaire for the veterinary professionals from Baghlan and surrounding provinces was also designed to assess their perception of the FMD situation in Baghlan and throughout Afghanistan, as most were experienced field practitioners. Two main questions (their perceptions about FMD and the failure to control FMD) were administered to individuals from this group. Answers were scored by participants from 1 to 12, as outlined previously.

## 3. Results

### 3.1. Knowledge of the Farmers toward Foot and Mouth Disease

The level of knowledge of farmers on FMD is summarised in Table 1. Most farmers (94.9%) obtained information about FMD from their village veterinarian or para-veterinarian. Almost half of the farmers (96; 48.5%) knew FMD from its name, and of 96, 46.9% of them had seen or heard of reports of cases of FMD in livestock in their village in the 12 months preceding the survey. Of the farmers who had observed FMD in their herd in the year preceding the survey, 44.4% reported that a larger number of outbreaks had occurred in spring than other seasons. The majority of farmers could correctly identify the clinical signs of FMD (between 77% and 86%).

### 3.2. Attitudes and Practices of Farmers towards Foot and Mouth Disease

The descriptive results for the responses to questions relating to farmers’ attitudes and practices are summarised in Table 2. Approximately two thirds of the farmers (63.1%) thought that the primary cause for the introduction of disease into their herd was through the introduction of new animals.

Most farmers (74.8%) reported that they would notify the local authorities if they suspected a case of FMD in their herd. To prevent and control the disease, nearly two-thirds of the participants (61.6%) thought that the intervention by the local veterinary authorities during the early stages of the disease was necessary. In order to minimise the risk of FMD introduction in their herds, approximately half (55.6%) of the farmers preferred not to buy cattle from unknown or potentially infected sources. Nearly all of the farmers (96.5%) were interested to learn more about FMD. A small number of farmers (18.7%) would use traditional methods of treating animals showing clinical signs (such as standing them in stream water or applying alum crystal locally known as “Zamj” to the infected area).

### 3.3. Local Animal Traders and Butchers’ Knowledge, Attitudes and Practices towards FMD

A total of 4735 sheep, 1715 goats, 490 cattle, and 65 buffalos are imported by the traders in the study area each year. Answers to questions by the local animal traders and the butchers (who are also considered animal traders) on FMD are summarised in Table 3. All 25 respondents had good general knowledge about FMD, although this was primarily limited to knowing the disease’s name and some of the clinical signs of the disease. According to the respondents, livestock imported into the study area from other regions of Afghanistan, including other districts in Baghlan province, were a major source of disease introduction into the study area. The majority of the ruminants (64%) imported into the study area were for meat purposes. Local consumers were ranked as most important (76%) for buying animals and animal products. All respondents (both traders and butchers) sourced animals only from within Afghanistan, which did not require any health certification from the authorities. No cases of FMD were reported by the ten butchers in the animals that they purchased for slaughter in the 12-month period prior to the questionnaire being administered. All animals purchased were slaughtered immediately or within a day of purchase. The majority (83.3%) of respondents mentioned that animals affected with FMD were sold at a lower price than healthy animals. If they observed a case of FMD during their trade, 91.7% of them would contact their local veterinarian. Only 8.3% had ever used traditional methods (as outlined in Section 3.2) to treat cases of FMD.

### 3.4. Local Veterinary Professionals’ Practices in the Study Area

Over half (*n* = 13, 65%) of the local veterinary professionals surveyed maintained record books to record details on the animal diseases seen and the animals treated in their area. All the record books used by the veterinary field units (VFUs) were provided by local NGOs (Table 4).

Most local veterinary professionals (*n* = 16, 80%) stated that they would report FMD cases to the authorities as a normal part of their work activity. A total of 646 cases of FMD were reported/recorded in the study region in the year preceding the survey, and of these cases 55.7% were in sheep, 39.7% cattle and 4.6% goats. All respondents used the same tetravalent or polyvalent vaccines against the three FMDV types previously detected by the government in the area. The two types of vaccine used were of Russian (FMD Vaccine adsorbed Polyvalent Liquid Inactivated vaccine containing A Iran 05, O Panasia 2, and Asia-1 types supplied in 25 or 50 dose bottles) or Turkish origin (Tetravalent, A Nep 84 (GVII), A Tur 16 (GII), O Tur 07, Asia 1 Tur 15 which was supplied in a 25-dose bottle).

### 3.5. Perception of Veterinary Professionals on FMD

The perceptions of experienced veterinarians and para-veterinarians from the surrounding provinces on FMD are summarised in Table 5. Uncontrolled seasonal movements of animals (90%), poor import controls and quarantine of live animals (including ruminants) and animal products (83.3%), direct contact between animals in free grazing areas (73.3%), lack of serotype-specific vaccines (60%) and the fast spread of the FMDV (56.7%) were considered to be the most important factors contributing to the endemic level of the disease in the study area, as well as across the country. A lack of community support, principally disease reporting by stakeholders, was the least (10%) concerning factor involved in the spread of FMD.

## 4. Discussion

Data on the knowledge, attitudes, and practices (KAP) of stakeholders can help in the planning, implementation, and evaluation of disease control programs. Furthermore, these are useful to identify knowledge gaps and cultural and behavioural differences between groups that may impair the success of a project [10]. The KAP questionnaires administered in the current study provided a valuable insight into the awareness and approaches of farmers towards FMD, in the study area. The study reported in this manuscript is the first documented research describing the KAPs for cattle farmers, animal traders, and veterinary professionals in Baghlan Province and Afghanistan. The data collected showed that FMD was a reasonably well-known disease among the cattle farmers, and they were well acquainted with traditional methods of raising animals and the treatment of animal diseases.

The study highlighted knowledge and attitude gaps which, if addressed, might assist in reducing or even eliminating the spread of FMD within the study area. Although only approximately half of the farmers (48.5%) had heard of or knew the name of the disease, a higher proportion of them recognised the characteristic clinical signs of the disease (between 77% and 86%). Almost similar findings have been reported in other parts of the world such as in Sri Lanka (farmers know clinical signs of FMD between 53% and 78%) and in Kenya (farmers know clinical signs of FMD between 53% and 78%) [11,12]. Others have similarly reported that livestock owners are often aware of the prominent clinical signs of the disease in both their own and neighbouring herds [13,14]. This is especially the case in cattle, where the clinical signs of FMD are more apparent [15,16] than in sheep and goats that show mild or no clinical signs of the disease [17]. The small number of livestock owned and the close association between farmers and their livestock increases the likelihood that the presence of a clinical condition, such as FMD, is detected or recognised [18]. The efficient knowledge on the clinical signs of FMD is related to the amount of information provided to livestock owners through multiple learning opportunities such as posters and passive information transfer [19] which also applies in the case of cattle farmers in the study area.

Farmers also reported that the animal movement, specifically introducing new animals to cattle herds, was believed to be the main reason for the entry of FMD into their cattle herds. It is widely accepted that the movement of infected animals is the most critical method for the spread of FMD, both within and between regions [20]. Often the virus initially enters a country or region by live animals or on contaminated products and then is distributed through livestock movement [21]. The current study results highlighted that farmers report diseases to the authorities when clinical signs of FMD are observed in their cattle. Immediate reporting of animal disease by the farmers is crucial for controlling and eliminating the disease by the local and regional authorities [22,23].

Only a few farmers stated that their susceptible animals were vaccinated against the FMDV, although this was only done once a year due to the unavailability of the vaccine(s) or vaccination programs by the authorities. Currently, there is no regular vaccination program conducted by the government or by NGOs in Baghlan province. This could be primarily due to the long-standing conflicts, making many locations unsafe to visit, the geographical remoteness of some locations which restrict the delivery of effective veterinary services to livestock [7,24], and the fact that many of the veterinary professionals in the country, specifically veterinary graduates, lack detailed knowledge and skills to perform basic veterinary activities [25]. In this study, it was also observed that some para-veterinarians and some of the new veterinary graduates in the study area were unable to collect blood, restrain cattle, or know how to store vaccines and veterinary medicines, diagnose a disease, or administer antibiotics correctly. Additionally, the availability of quality vaccines and transportation of vaccines and other veterinary medicines is challenging in Baghlan province. Others [25] have also highlighted the inefficiency of veterinary services, specifically concerning the widespread availability of poor-quality medicines and incorrectly stored/expired animal vaccines at local markets throughout Afghanistan. However, 20% of the interviewed veterinary professionals advised that they regularly vaccinated animals (at least once a year-depending on the availability of vaccines) in their region. Despite considerable awareness about FMD by the farmers, the endemic level of the disease and the lack of an organized vaccination campaign means the disease remains a major threat to livestock in Baghlan province. One of the main limitations to eradicating FMD is the lack of quality vaccines that boost the immune system of the animal against clinical signs and prevent infection [26]. The importance of vaccination against FMDV was highlighted by Domingo, Baranowski [27], Carrillo, Wigdorovitz [28], who reported that vaccinating all susceptible animals against FMDV with the inactivated virus had been very successful in controlling and preventing the disease in different countries and regions of the world, including Thailand [29] and Europe [30]. It is considered that the best method for the control and future eradication of FMD in herds within the study area is the implementation of emergency vaccination. This has been identified by Keeling, Woolhouse [31] as one of the most successful strategies to control FMD in an area when localized outbreaks occur. Although the control and eradication of FMD in an area can be achieved through implementing a stamping out program [32]. This is often inappropriate in countries/areas where the disease is endemic and results in high costs and loss of livestock for human consumption [32]. Currently, eradication would not be appropriate for Afghanistan as a low-income country due to the limited resources available. However, alternative control measures (e.g., vaccination and quarantine) are preferred.

In the current study, it was also observed that farmers tended to avoid buying cattle from risky sources (unknown areas/markets or areas with previous reports of FMD). Others [33,34] also identified sale-yards (markets) as potential risks for the fast spread of FMD in a region, particularly in the case of Afghanistan where the movements of animals are not controlled and animals are in close contact with animals from other herds/flocks in sale-yards [35]. Similarly, Bhattacharya, Banerjee [36] also reported the role of unrestricted movement of animals in the animal markets in the spread of FMD in West Bengal, India.

One of the typical local treatments of lesions in infected cattle was the application of alum crystals (potassium aluminum sulfate “KAl(SO_4_)_2_·12H_2_O” known as Zamj and patrakai in the local languages of Dar/Farsi and Pashto) on lesions. This is a traditional treatment, also called Unani Medication (Greek Medication), and is widely used in Iran, Afghanistan, Pakistan, and India [37]. The use of alum for treating lesions in the mouth of sick animals has also been reported by others [37,38] as it cauterises the vesicles and erosions present. Another traditional treatment commonly used by the surveyed farmers and animal traders was to stand the infected animal in a stream or river to clean foot lesions and to decrease the pain associated with these lesions. This method offers the advantage of relieving the discomfort associated with open ulcers/erosions. However, it potentially could be a risk factor for the spread of FMDV in endemic regions through releasing the virus into the water or through the mixing of livestock at such locations [39]. In a similar study by Sieng, Patrick [14] it was found that farmers are using various traditional methods to treat their sick cattle such as: using herbs and engine oil aimed to clean lesions and deterring flies, and walking infected cattle through the mud to cauterise the pain arising from lesions.

In the study area, local veterinary professionals are the leading source of information for cattle farmers, which indicates a strong interaction between the farming community and the local veterinary authorities. Others [40,41,42] have also highlighted the importance of local veterinarians as one of the leading and trusted sources of health management and biosecurity information for livestock farmers.

Like farmers, animal traders in Baghlan province had a good general knowledge about FMD and its associated clinical signs, although their perceptions of controlling FMD were weak compared to farmers. However, importantly, during the trade of animals, veterinarians were the main point of contact when FMD cases were observed, even though the majority of traders (83.3%) preferred to sell sick animals that were displaying the early stages of clinical disease at a discounted price, rather than treating these animals. Livestock traders are an essential stakeholder in public awareness campaigns, and the need for communication between veterinary authorities and traders is as vital as it is for farmers [43]. It was observed that traders in Baghlan province purchased cattle from remote areas and subsequently sold them in the livestock markets located in the province’s districts. The livestock markets used by the traders are an “open system”, where large numbers of different species of animals from different regions (districts within the province, as well as from surrounding provinces) are mixed and traded. The traders typically purchased cattle, resold them quickly (within one day to a week), and had no intention of keeping purchased animals for a long time. This was likely to result in the rapid distribution of livestock throughout the districts and provinces of Afghanistan and potentially facilitate the rapid spread of FMDV.

In this study, it was observed that local veterinary professionals had been actively participating in disease investigations and conducting passive surveys by reporting FMD cases in their local villages through the help of local and regional NGOs. Disease surveillance is a key function for animal health management [44,45]. In the case of FMD surveillance in the study area, if local veterinary professionals increase their activities, they will establish a strong communication bond between the veterinary authorities and the farmers while collecting and analysing data from cattle herds in their work area. Adopting such activities offers benefits for farmers and the livestock industry that are not just restricted to the control of FMD [46,47]. Communication between farmers and veterinarians has been identified as a necessary tool to improve farm management [48,49]. One of the many benefits of the ongoing communication between veterinarians and dairy farmers is that the activities of veterinarians are being transferred from task-oriented providers of single-cow therapy to advice-oriented herd health management advisors [49]. Veterinarians working in close contact with farmers can address food safety, public health, animal health, and welfare in the whole production process on the dairy farms [50]. This advisory approach by the veterinarian should result in significant positive outcomes for the health of dairy cows and reduce the economic burden of disease on the farmers and consumers.

Collecting information on the perceptions of experienced veterinary professionals’ on FMD within the study area and nationally in Afghanistan was a crucial part of this survey. These veterinary professionals identified concerns over the government’s inability to restrict animal movements, impose quarantine measures at border points (provincial and national borders), and define disease zones. These concerns, along with the absence of a national animal disease database, were considered to be linked to the failure to control and eradicate FMD in the country. The movement of animals within communities or between countries has been considered the leading factor for the dispersal of FMDV nationally and globally [13,51,52,53]. Others [54,55] have also pointed out the inability of veterinary authorities to control animal diseases in Afghanistan. In particular, a lack of well-defined veterinary treatment activities of the veterinary department, inability to control zoonotic diseases, an absence of active disease surveillance activities, and the absence of quality control of vaccines and medicines were identified as factors preventing effective disease control.

Although this survey collected valuable information, there were potential limitations in its application. Therefore, the interpretation of the results arising from it. Farmers potentially may have had recall bias on the occurrence of outbreaks, and many were unable to remember the exact date or season of the last outbreak of FMD in their farms or villages. Similarly, farmers often could not recall the exact type of medication and vaccines administered to their cattle. Even though most of the farmers were well-aware of the damages caused by FMD, some were reluctant to provide detailed information about it, potentially due to the lack of ongoing communication with government authorities and the absence of a regular control program implemented by the government. Some farmers also did not have a basic knowledge of hygiene to adopt in their cattle herds, despite raising cattle for many decades and having close communication with veterinary professionals.

Other challenges were also evident during this study. The care and maintenance of veterinary medicines and animal vaccines were poor, primarily due to a lack of regular electricity supply at most VFUs. Although it was observed that farmers would immediately report clinical signs of FMD to the local veterinary authorities, the reporting system within the veterinary authorities in the area was inefficient and dependent on financial assistance from NGOs and the government. Others [7,55] also have highlighted that the disease reporting system within the veterinary activity framework in Afghanistan is limited or even non-existent, which leads to deficiencies in submitting samples to reference laboratories [56], resulting in the ineffective implementation of control measures within the area. The ongoing war and security issues in some districts of Baghlan province and the surrounding provinces are also challenges that prevent the veterinary authorities from providing an effective service to the local farmers and livestock industries. These constraints to the livestock sector in Afghanistan, particularly within the study area, prevent the livestock sector from contributing effectively to international trade, with the development of the livestock industry driven by unpredictable aid (Rushton [44]).

In conclusion, this study on the KAPs of farmers, traders, and veterinary professionals provided helpful information on FMD, which could be used to improve the future implementation of methods to control FMD within the region. Both farmers and animal traders were aware of FMD and could identify cases of the disease based on the presenting clinical signs. One significant observation of this survey was that the farmers were willing for their cattle herds to be vaccinated regularly if quality vaccines were available. It was also observed that farmers were motivated to increase their knowledge about the disease, specifically its prevention and control. They also expressed willingness to report cases of the disease. Furthermore, there is a need to improve interprovincial quarantine, identify movement patterns of animals, minimize movements of cattle, and ascertain the social impact of FMD to develop an effective control/eradication program for the disease in Baghlan Province.

## Figures and Tables

**Table 1:** Knowledge and awareness of 198 farmers from Baghlan Province, Afghanistan, about foot and mouth disease (FMD).

Knowledge Item	Frequency	Percentage (95% CI)
Farmers’ source of information about FMD		
Village veterinarian or para–veterinarian	188	94.9 (90.9–97.6)
Radio	49	24.8 (18.9–31.4)
TV	27	13.6 (9.2–19.2)
Village or community leaders	14	7.1 (3.9–11.6)
Other sources (neighbors, relatives, and friends)	5	2.5 (0.8–5.8)
Brochures/Posters (provided by government or NGOs)	4	2 (0.6–5.1)
Livestock wholesalers or traders	3	1.5 (0.3–4.4)
Newspaper	1	0.5 (0.0–2.8)
Respondents had seen or known the clinical signs of FMD (farmers shown photographs of lesions)		
Excess salivation	170	85.9 (80.2–90.4)
Ulcers on the tongue	156	78.8 (72.4–84.3)
Lesions on the gums	155	78.2 (71.9–83.8)
Lesions on the hooves	152	76.8 (70.3–82.5)
Lesions on the udder &/or teats	28	14.1 (9.6–19.8)
Respondents had heard of FMD in their village (anytime in the past)		
No	102	51.5 (44.3–58.7)
Yes	96	48.5 (41.3–55.7)
The clinical signs of FMD were observed by farmers in the most recent outbreak seen by them		
Ulcers on the tongue of cattle	91	94.8 (88.3–98.3)
Salivation in cattle	91	94.8 (88.3–98.3)
Lesions on the gums of cattle	88	91.7 (84.2–96.3)
Lesions on the hooves of cattle	87	90.6 (82.9–95.6)
Lesions on the udder and teat of cattle	4	4.2 (1.1–10.3)
Respondents had observed cases of FMD in their village in the 12 months immediately preceding the questionnaire		
No	51	53.1 (42.7–63.4)
Yes	45	46.9 (36.6–57.3)
The season when FMD cases were noticed in the last 12 months		
Spring (21 March–21 June)	20/45	44.4 (29.6–60)
Didn’t remember	10/45	22.2 (11.2–37.1)
Summer (22 June–22 September)	9/45	20 (9.6–34.6)
Autumn (23 September–21 December)	5/45	11.1 (3.7–24.1)
Winter (22 December–20 March)	1/45	2.2 (0.1–11.8)

**Table 2:** Farmers’ (*n* = 198) attitudes and practices towards control of foot and mouth disease (FMD) in their herds.

Attitudes	Frequency	Percentage (95% CI)
The potential source of FMD for their herds:		
Introduction of new animals	125	63.1 (56.0–69.9)
Neighbouring herds	74	37.4 (30.6–44.5)
People, equipment & vehicles entering from infected sources	62	31.3 (24.9–38.3)
Contaminated feed	17	8.6 (5.1–13.4)
Other sources (example: dog bringing part of a carcass into an area where cattle are kept)	6	3 (1.1–6.5)
What would you do if you suspected that your herd had FMD?		
Report immediately to authorities	148	74.8 (68.1–80.6)
Treat the affected animals myself (including using traditional methods)	37	18.7 (13.5–24.8)
Do nothing	18	9.1 (5.5–14)
Sell the cattle to the village butcher	9	4.6 (2.1–8.5)
Other	6	3 (1.1–6.5)
Slaughter cattle for meat	4	2 (0.6–5.1)
What do you see is necessary to prevent or control FMD?		
Early FMD detection by local veterinary professionals	122	61.6 (54.5–68.4)
Regular visits by the veterinary authorities	78	39.4 (32.5–46.6)
Safe source of new animals introduced to herd/village	42	21.2 (15.7–27.6)
Control of infected animal movements by the authorities	30	15.1 (10.5–20.9)
Reduce contact between my herd and other herds	28	14.1 (9.6–19.8)
Provide compensation for farmers to cull their infected animals	26	13.1 (8.8–18.6)
Provide clean feed and water for their herd	16	8.1 (4.7–12.8)
Other	7	3.5 (1.4–7.1)
How are you currently protecting your herd from getting FMD?		
Not buying cattle or other ruminants from risky sources	110	55.6 (48.3–62.6)
Not doing anything	43	21.7 (16.2–28.1)
Other	33	16.7 (11.8–22.6)
Disinfecting animal stable regularly	23	11.6 (7.5–16.9)
Ensuring clean water and feed are given to my animals	18	9.1 (5.5–14)
Are you interested in receiving further information on FMD?		
Yes	191	96.5 (92.9–98.6)
No	7	3.5 (1.4–7.1)
What specific information on FMD would you like to know?		
Method of preventing the disease	168	84.9 (79.1–89.5)
How to treat affected animals	82	41.4 (34.5–48.6)
Basic knowledge about the disease	26	13.1 (8.8–18.6)

**Table 3:** Traders’ (*n* = 15) and butchers’ (butchers in Baghlan province purchased live animals for their business, therefore they were also considered as animal traders.) (*n* = 10) responses related to their knowledge and attitudes on foot and mouth disease (FMD).

Knowledge and Attitudes	Frequency/Number	% (95% CI)
For what purpose do you purchase animals (cattle)?		
Meat	16	64 (42.5–82.0)
Milk	6	24 (9.4–45.1)
Did not provide an answer	2	8 (1.0–26.0)
Breeding	1	4 (0.1–20.4)
Who do you usually sell your animals to?		
Local consumers	19	76 (54.9–90.6)
Farmers	15	60 (38.7–78.9)
Butchers	14	56 (34.9–75.6)
Other traders	8	32 (14.9–53.5)
Others (contracts to government deps)	1	4 (0.1–20.4)
Do you purchase/import animals with a health certificate?		
No	25	100 (86.3–100)
Yes	0	0
Have you ever had an outbreak of FMD in livestock you purchased?		
No	13	52 (31.3–72.2)
Yes	12	48 (27.8–68.7)
Has it ever happened that you have had animals infected with FMD that no one was interested in buying?		
Yes	9	75 (42.8–94.5)
No	3	25 (5.5–57.2)
Have you ever had any infected animals die from FMD?		
Yes	1	8.3 (0.2–38.5)
No	11	91.7 (61.5–99.8)
How did FMD affect your livestock?		
Animals were sold for a cheaper price than non–affected animals	10	83.3 (51.6–97.9)
Animals were treated	1	8.3 (0.2–38.5)
Animals died	1	8.3 (0.2–38.5)
What did you do when your animals were affected with FMD?		
Call the local veterinarian	11	91.7 (61.5–99.8)
Use traditional treatment(s)	1	8.3 (0.2–38.5)

**Table 4:** Local Veterinary professionals’ profile activities on foot and mouth disease (FMD) in the study area.

Veterinary Professionals Profile		Number of Respondents
Level of education	Veterinarian	10
	Para-veterinarian	8
	Masters in veterinary science	1
	Basic Veterinary Worker	1
Years of experience in the field	1–<5 years	4
	5–10 years	5
	>10 years	11
VFU funded by	Private	14
	Non-Government Organisation	6
**Practice item**	**Frequency**	**Percentage (95% CI)**
Do you have a record keeping book?		
Yes	13	65 (40.8–84.6)
No	7	35 (15.4–59.2)
Do you record all diseases reported to you, including FMD?		
Yes	13	100 (75.3–100)
No	0	0
Do you have records of FMD cases for the year preceding the survey?		
Yes	13	100 (75.3–100)
No	0	0
Do you have historical records of cases of FMD from more than one year ago?		
Yes	11	84.6 (54.6–98.1)
No	2	15.4 (1.9–45.4)
What species of animals and how many of them were involved in the most recent outbreak of FMD?		
Sheep	360	55.7 (51.8–59.6)
Cattle	256	39.6 (35.8–43.5)
Goats	30	4.6 (3.2–6.6)
Who do you report to if you have an outbreak of FMD?		
Agha Khan Foundation	6	37.5 (15.2–64.6)
Provincial Veterinary Office	5	31.3 (11.0–58.7)
Dutch Committee for Afghanistan	4	25.0 (7.3–52.4)
I don’t report the outbreak to anyone	4	25.0 (7.3–52.4)
Regional Veterinary Office	1	6.3 (0.2–30.2)
What type of diagnosis is made at the local Veterinary Field Units?		
Clinical diagnosis	20	100 (83.2–100)
Do you have a FMD vaccination program in your area?		
Yes	6	30.0 (11.9–54.3)
No	14	70.0 (45.7–88.1)
How often do you vaccinate animals against FMD in your area?		
Every six months	4	66.7 (22.3–95.7)
Every twelve months	1	16.7 (0.4–64.1)
When there is an outbreak around the area	1	16.7 (0.4–64.1)
What type of vaccination program do you follow in your area?		
Protective (to protect groups of animals from infection or clinical signs of disease (includes ring, targeted and buffer vaccination strategies).)	3	15.0 (3.2–37.9)
Suppressive (to control the spread of FMD within and out of an infected area by vaccinating selected groups of animals.)	17	85.0 (62.1–96.8)
What type of FMD vaccine do you use?		
Trivalent and Tetravalent (one type of tetravalent vaccine (Turkish origin) was available in the market and used for three detected subtypes in the province and the country)	20	100 (83.2–100)

**Table 5:** The perceptions of the veterinary professionals (*n* = 30) about foot and mouth disease (FMD).

Respondents’ Profile		Number of Respondents		
Level of education	Veterinarian	21		
	Para-veterinarian	6		
	Master’s in veterinary and Animal Sciences	2		
	Basic Veterinary Worker (BVW)	1		
Years of experience on the field	1–<5 years	7		
	5–10 years	14		
	>10 years	9		
Veterinary Field Unit funded by:	Private	11		
	Government	10		
	Non-Governmental Organization	9		
Q1. What are the main barriers to the control or prevention of outbreaks of FMD in Afghanistan?				
**Factors**		**Percentage of Respondents**		
		**Most Important**	**Moderately Important**	**Least Important**
Uncontrolled seasonal movement of animals in the country		90	6.7	3.3
Poor import controls and quarantine		83.3	10	6.7
Direct contact between animals in free grazing		73.3	16.7	10
Lack of serotype-specific vaccines		60	26.7	13.3
The fast spread of the FMDV		56.7	23.3	20
The short-term immunity induced by the vaccines		10	46.7	43.3
Absence of cross-immunity between the seven serotypes of FMDV		10	40	50
Lack of good hygiene and sanitary practices		6.7	53.3	40
Lack of community support (disease reporting by stakeholders, farmer’s reluctance, poverty resulting in the inability to pay for treatment.)		6.7	3.3	90
Q2. What do you believe hinders the eradication and control of FMD in Afghanistan?				
**Factors**		**Ranking Percentage**		
		**Most Important**	**Moderately Important**	**Least Important**
Absence of appropriate legal powers in the Veterinary Department		63.3	23.3	13.3
Lack of well-defined zones (infected zones, surveillance zones, FMD-free zones)		50	33.3	16.7
The absence of the Animal Disease Database System (ADDS) to have a record of regular and prompt animal disease reporting.		50	20	30.0
Lack of well-trained personnel (vaccination team, etc.) and access to necessary financial and other resources (equipment, materials, etc.)		46.7	13.3	40
Lack of security and stability in the country		36.7	23.3	40
Lack of stamping out programmes for rapid eradication of FMD		33.3	36.7	30
Lack of capabilities to stamp out infected animals and compensate farmers		26.7	40	33
Lack of assistance with veterinary authorities from agencies such as: police, defence force, ministry of labour and social affairs, media		26.7	30	43.3
Lack of knowledge about the circulating FMD serotypes and strains throughout the course of vaccination campaign		20	23.3	56.7
Lack of political commitment to control FMD and FMD type infections		16.7	43.3	40
Lack of accurate serological tests in government laboratories		6.7	26.7	66.7
Lack of disease surveillance systems to monitor the effectiveness of vaccination and to detect remaining pockets of infection		6.7	13.3	80

## Data Availability

All data supporting the reported results are presented in the article.
